# Robust Input Shaping Commands with First-Order Actuators

**DOI:** 10.3390/mi15091086

**Published:** 2024-08-28

**Authors:** Yoon-Gyung Sung, Seongjun Lee

**Affiliations:** Department of Mechanical Engineering, Chosun University, 309 Pilmun-daero, Dong-gu, Gwangju 61452, Republic of Korea; sungyg@chosun.ac.kr

**Keywords:** robust input shaper, first-order actuator, phase vector diagram, flexible systems

## Abstract

This paper presents robust input shaping commands with first-order actuators utilizing a classical robust input shaper for practical applications in input shaping technology. An ideal input shaping command can deviate due to actuator dynamics so that the modified command has a detrimental effect on the performance of oscillation reduction in feedforward control applications. A zero-vibration-derivative (ZVD_F_) shaper with first-order actuators is analytically proposed using a phasor–vector approach, an exponential function for the approximation of the dynamic response of first-order actuators and the usage of the ZVD shaper. In addition, an equivalent transformation is utilized based on the superposition principle for the convenient inclusion of first-order actuator dynamics and is applied to the individual segment input command. The residual deflection and robustness of the proposed robust input shaping commands are numerically evaluated and compared with those of a conventional ZVD shaper with respect to the parameter uncertainties of flexible systems and actuators. The robust input shaping commands that are possible with first-order actuators are experimentally validated, presenting a better robustness and residual deflection reduction performance than the classical ZVD shaper on a mini bridge crane.

## 1. Introduction

Input shaping technology is an effective method for eliminating residual oscillation in point-to-point maneuvers of linear or nonlinear flexible systems [1,2,3,4]. In addition to the nonlinear characteristics of structural systems, the performance of input shaping methods can be significantly degraded by the collapse of command profiles attributed to actuator dynamics. Therefore, it is necessary to develop a robust input shaping technique analytically without extending the duration to account for actuator dynamics.

Several analytically robust input shapers have been proposed. As an initial input shaper to overcome system modeling errors, the zero vibration (ZV) shaper was shown to be effective under system parameter variations. To ensure the robustness to modeling errors, a zero-vibration derivative (ZVD) shaper was proposed by setting the derivative of the residual oscillation amplitude of a second-order system to the impulse sequence input with respect to the natural frequency [5,6,7]. To further improve insensitivity, the differentiation process can be repeated by taking into account additional higher-order derivatives with respect to the natural frequency. The cost of each additional derivative is an increase in the shaper duration by half of the period of the natural frequency [8]. While maintaining the same number of impulses as in the ZVD shaper, an EI (extra insensitive) shaper was proposed by relaxing the zero-residual oscillation constraint to a tolerable level. The EI shaper has the same impulse time locations as the ZVD shaper but has different amplitude values that lead to the advantage of improved robustness [9]. A specified insensitivity (SI) shaper was presented for system modeling errors by extending the formulation of the EI shaper to an arbitrary specified oscillation level [10]. The SI shaper indicated that the residual oscillation magnitude was within the specified tolerance level. Numerous input shapers that add impulses have been proposed; however, an increase in the duration of shaping commands that cope with modeling errors is inevitable.

Nonlinear actuator dynamics can significantly influence residual oscillation. Nonlinear dynamics can be caused by discontinuous nonlinearities in a system such as backlash, dead-zone, saturation, rate-limiting, and electronic nonlinear dynamics [11]. Owing to the nonlinear actuator, shaping commands can be modified such that the performance of oscillation reduction degrades drastically. To mitigate the adverse effect of deflection reduction performance from the perspective of backlash and Coulomb friction, a nonlinear input shaping command was presented by creating negative impulses of the on–off type considering the nonlinear actuator’s characteristics [12,13,14]. For the nonlinearities of the dead-zone and backlash, an inversion technique was proposed [15,16] with an analysis of the uncertainties in the dead-zone and backlash width. Classical input shaping techniques have been applied to systems with nonlinearity arising from the asymmetrical acceleration of the actuator of a bridge crane [17,18]. The effect of a nonlinear actuator on the implementation of a unity-magnitude (UM) ZV input shaper was minimized by updating the temporal locations of the impulses of the shaper to minimize residual crane vibrations. To constrain the transient and residual deflections during the rest-to-rest operation of flexible systems, a deflection-limiting input (DLF) shaper with first-order actuators was developed using the vector diagram approach. The DLF shaper exhibited better control performance than a conventional input shaper [19]. Without the use of linearization and a greater impulse sequence, closed-form ZVD and EI shapers with rate-limiting actuators using the phase vector approach, ramp-step approximation function, and classical input shapers have been presented [20]. Two robust input shapers presented a much better residual deflection reduction performance than classical robust input shapers with respect to the uncertainty of the system modeling and actuator parameters. Therefore, nonlinear actuator dynamics have an adverse effect on feedforward control and are considered to improve the deflection reduction performance of the input shaping technique.

In this study, we propose robust input shaping commands that consider the nonlinear dynamic characteristics of actuators in real industrial fields. In Section 2, the proposed zero vibration derivative first-order (${ZVD}_{F}$) shaper is established by applying the vector diagram approach to the phasor form through the steady-state response equation of a pendulum system while considering the dynamic characteristics of the nonlinear actuator. A closed-form solution is presented by utilizing the impulse amplitudes of ZVD input shaper. In Section 3, the performance of the ${ZVD}_{F}$ shaper is numerically evaluated with respect to the residual deflection and robustness. In Section 4, the sensitivity and residual oscillation performance of the proposed robust shaper are evaluated experimentally using a mini bridge crane.

## 2. Robust Input Shaper with First-Order Actuators

A robust input shaper with first-order actuators was developed analytically using three impulse sequences. In the development process, the impulse magnitudes of a conventional robust input shaper were utilized to determine the impulse time locations using the phase vector approach and equivalent command transformation [11]. In addition, an exponential function was exploited for the approximation of the distorted shaped command, owing to the first-order actuators.

**Figure 1:**
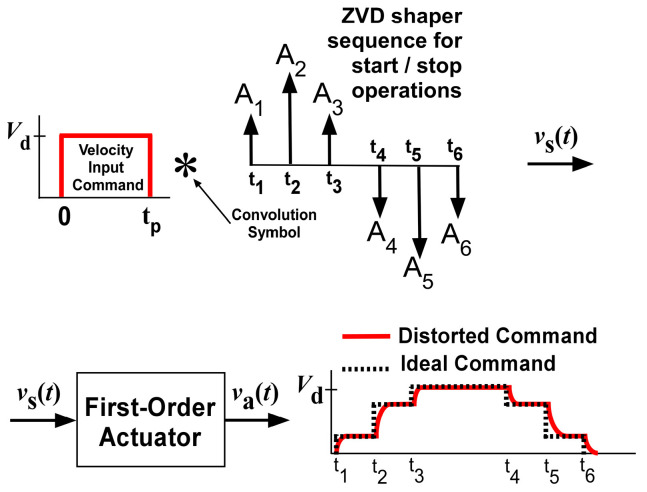
Actuator effects on a ZVD shaper.

In ideal actuators, ideal-shaped input commands are generated by convolving the pulse input and three impulse sequences only for linear systems to maintain the robust oscillation reduction performance. However, a first-order actuator can distort the shaped command, as shown in Figure 1. Distorted shaped commands can drastically degrade the deflection reduction performance during the operation of flexible systems. Therefore, the development of a robust input shaping command using a first-order actuator is required.

**Figure 2:**
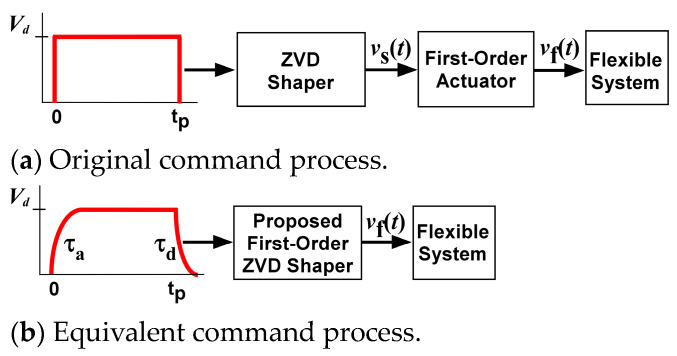
Equivalent transformation.

The ${ZVD}_{F}$ shaper was developed based on a ZVD shaper. The ZVD shaper is briefly summarized as follows [5]:

(1) $$[\begin{array}{c} A_{i}\\ t_{i} \end{array}]=\left[\begin{array}{ccc} \frac{1}{K^{2}+2K+1} & \frac{2K}{K^{2}+2K+1} & \frac{K^{2}}{K^{2}+2K+1}\\ 0 & ∆T & 2∆T \end{array}\right],\;i=1,2,3$$

where $∆T=\pi /(\omega_{n}\sqrt{1-\zeta^{2}})$ and K = $e^{-\zeta \pi /\sqrt{1-\zeta^{2}}}$ are expressed where $\omega_{n}$ and ζ are the natural frequency and the damping ratio of second-order systems, respectively. The ${ZVD}_{F}$ shaper is designed with a damping ratio of zero. The command design procedure was simplified with the transformation into an equivalent input command, as shown in Figure 2b, with acceleration time constant $\tau_{u}$ and deceleration time constant $\tau_{d}$. Using this approach, the shaper development was reformulated to determine an input shaper for the distorted input command. There was also the assumption that $t_{p}>3\tau_{a}$ so that there would be sufficient time to reach the desired velocity magnitude $V_{d}$.

**Figure 3:**
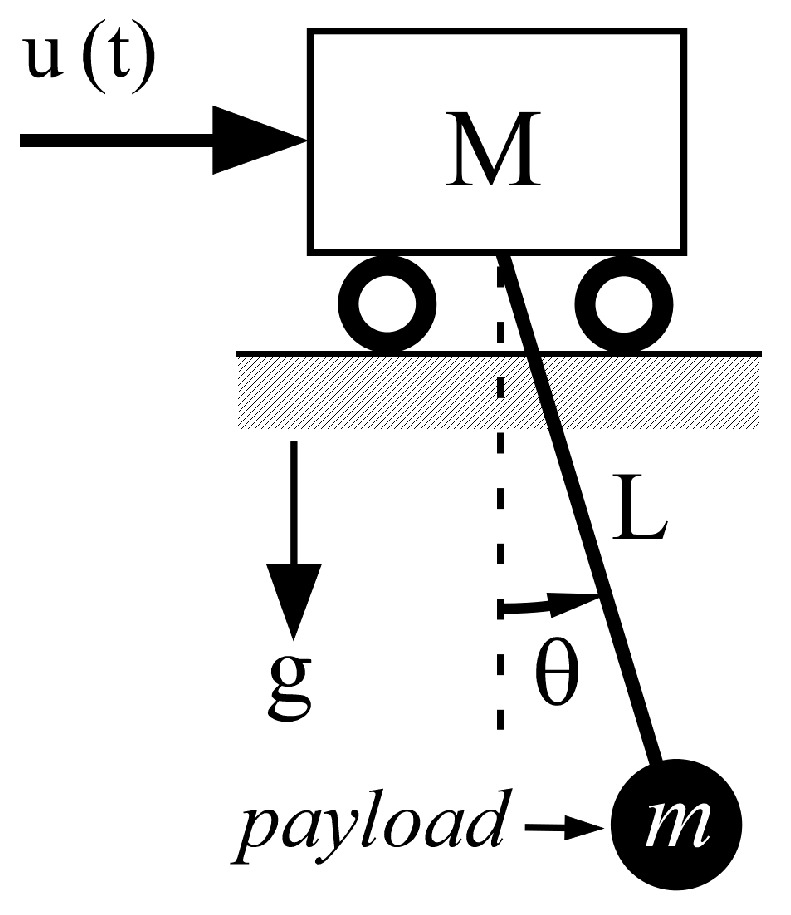
Pendulum system.

A pendulum system was used to propose an analytical solution for the development of a robust input shaper, as shown in Figure 3. The equation of motion is expressed as follows:

(2) $$L\ddot{\theta }\left(t\right)+g\sin\theta (t)=u\left(t\right)\cos\theta (t)$$

where $u(t)\;=v_{a}(t)$ is the velocity input command; L is the cable length; and g is the gravitational constant. With the assumption of a small angle Θ(t), we can express (2) using the Laplace transform as:

(3) $$\Theta (s)=G_{p}(s)V_{a}(s)=-\frac{{sV}_{a}(s)}{L}\frac{1}{s^{2}+{\omega_{n}}^{2}},$$

where $\omega_{n}$ is the natural frequency of the pendulum system and $V_{a}(s)$ is the Laplace transform of $v_{a}(t)$. The system output Θ(s) is redefined as

(4) $$\Theta (s)=\underset{H(s)}{\underbrace{-\frac{s.V_{a}(s)}{L\omega_{n}}}}.\underset{Sine\;input}{\underbrace{\frac{\omega_{n}}{s^{2}+{\omega_{n}}^{2}}}}.$$

Note that the second term in (4) is the Laplace transform of a sine wave with $\omega_{n}$ so that H(s) become a new transfer function. Because of the primary interest in residual oscillations, the development process of the input shaping method begins with the steady-state response payload response to a single step input derived by setting $s=j\omega_{n}$ and evaluating the phase and magnitude of $H\left(s\right)$ in (4) [21] as

(5) $$\theta_{ss}(t)=\frac{\left|V_{a}(j\omega_{n})\right|}{L}sin(\omega_{n}t-\frac{\pi }{2}+∠V_{a}(j\omega_{n})).$$

Equation (5) can be compactly expressed as a vector or phasor notation:

(6) $$\bar{v}=\frac{\left|V_{a}(j\omega_{n})\right|}{L}∠V_{a}(j\omega_{n})$$

This vector notation could be utilized as a graphic means of measuring the oscillation with the magnitude and the angle on a polar plot.

**Figure 4:**
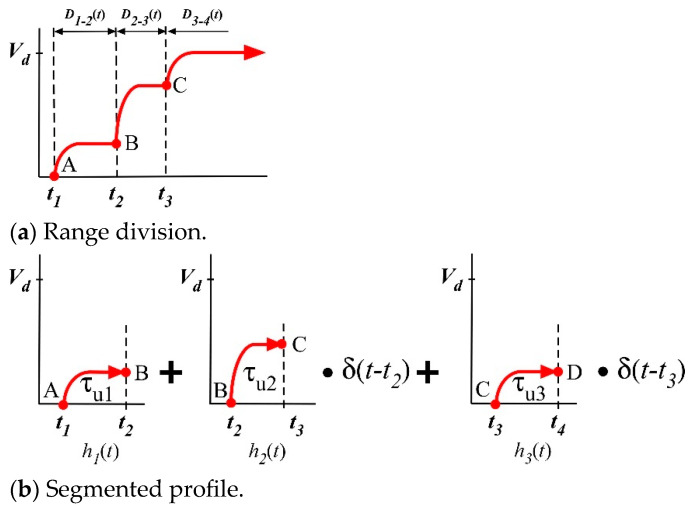
Segmentation of a start command.

The result of Equation (6) for a single step input is applied to a multi-step command generated by the convolution of input shaper and a step input as in Figure 4a. As shown in Figure 1, residual oscillations are produced from the start and stop periods of a distorted input command. For no residual oscillation from the start period of input command in Figure 4a, the magnitude of $\theta_{ss}(t)$ should be zero. Hence, $\left|V_{a}(j\omega_{n})\right|$ can be determined by the command division with respect to the impulse time locations of an input shaper, as shown in Figure 4b. The entire velocity profile is expressed as follows:

(7) $$v_{a}\left(t\right)=\sum_{i=1}^{3}h_{i}\left(t\right)\delta (t-t_{i}),$$

where $h_{i}(t)$ is a function of each region, as shown in Figure 4. Hence, an exponential function was employed to approximate the nonlinear dynamics of the actuator as follows:

(8) $$h_{i}(t)=A_{i}V_{d}(1-e^{-\frac{t}{\tau_{i}}}),$$

where $A_{i}$ is the impulse magnitude, $V_{d}$ is the desired velocity of a given actuator, and $\tau_{i}$ is the time constant in the start period of distorted input command. Each time constant in acceleration region was selected by reflecting the intermediate step magnitude variations with $\tau_{u1}$ after $t_{1}$, $\tau_{u2}$ after $t_{2}$ and, $\tau_{u3}$ after $t_{3}$. To determine the impulse time locations of the ${ZVD}_{F}$ shaper, a phase vector approach was utilized to set the vector sum to zero with no residual oscillation. Here, (7) was transformed by the Laplace transform and expressed as the vector magnitude and phasor as follows:

(9) $${\bar{v}}_{i}=\frac{\left|V_{i}(j\omega_{n})\right|}{L}∠\left[\omega_{n}t_{i}-\frac{3}{2}\pi +V_{i}(j\omega_{n})\right]$$

$$|V_{i}(j\omega_{n})|=\frac{A_{i}\;V_{d}}{\omega_{n}\sqrt{(\tau_{i}\omega_{n}{)}^{2}+1}},$$

$$∠V_{i}(j\omega_{n})={tan}^{-1}(\frac{1}{\tau_{i}\omega_{n}}).$$

Using the vector form of steady-state response in (9), the command vector of each region is obtained as

(10) $${\bar{v}}_{1}=|\frac{A_{1}V_{d}}{L{\;\omega }_{n}\sqrt{(\tau_{u1}\omega_{n}{)}^{2}+1}}|∠[\omega_{n}\;t_{1}-\frac{3}{2}\pi +{tan}^{-1}(\frac{1}{\tau_{u1}\omega_{n}})],$$

(11) $${\bar{v}}_{2}=|\frac{A_{2}V_{d}}{L\omega_{n}\sqrt{(\tau_{u2}\omega_{n}{)}^{2}+1}}|∠[\omega_{n}t_{2}-\frac{3}{2}\pi +{tan}^{-1}(\frac{1}{\tau_{u2}\omega_{n}})],$$

(12) $${\bar{v}}_{3}=|\frac{A_{3}V_{d}}{L\omega_{n}\sqrt{(\tau_{u3}\;\omega_{n}{)}^{2}+1}}|∠[\omega_{n}t_{3}-\frac{3}{2}\pi +{tan}^{-1}(\frac{1}{\tau_{u3}\omega_{n}})].$$

A closed-form ${ZVD}_{F}$ shaper can be obtained with the zero sum of all the three vectors by forming a closed triangle. In addition, the impulse magnitudes ($A_{1}$ = 0.25, $A_{2}$ = 0.5, and $A_{3}$ = 0.25) are assumed to be the amplitudes of the ${ZVD}_{F}$ shaper for a simplified formulation and no parameter optimization. By normalizing each command vector with respect to (10), the phasor vectors were expressed as

(13) $${\bar{v}}_{1}=|1|∠[0],$$

(14) $${\bar{v}}_{2}=|\mu |∠[\omega_{n}t_{2}-{tan}^{-1}(\frac{1}{\tau_{u2}\omega_{n}})+{tan}^{-1}(\frac{1}{\tau_{u1}\omega_{n}})],$$

(15) $${\bar{v}}_{3}=|\lambda |∠[\omega_{n}t_{3}-{tan}^{-1}(\frac{1}{\tau_{u3}\omega_{n}})+{tan}^{-1}(\frac{1}{\tau_{u1}\omega_{n}})],$$

where

$$\mu =\;\frac{2\sqrt{(\tau_{u1}\omega_{n}{)}^{2}+1}}{\sqrt{(\tau_{u2}\omega_{n}{)}^{2}+1}}$$

$$\lambda =\frac{\sqrt{(\tau_{u1}\omega_{n}{)}^{2}+1}}{\sqrt{(\tau_{u3}\omega_{n}{)}^{2}+1}}$$

**Figure 5:**
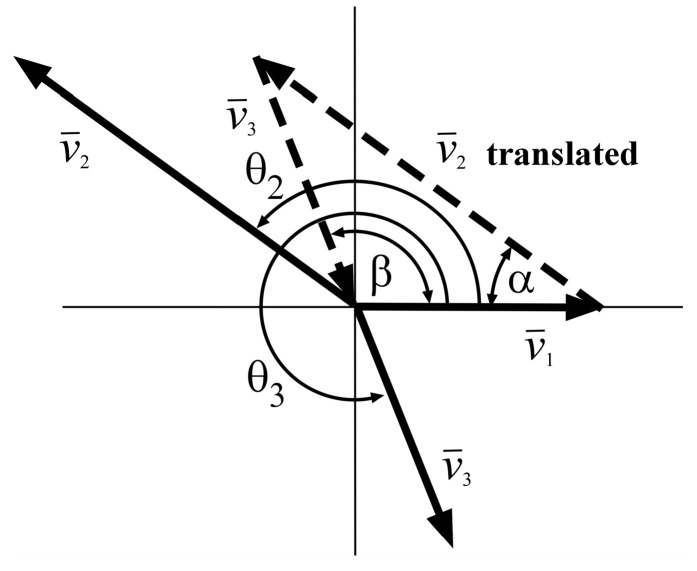
Vector diagram of a ZVD_F_ shaper.

As shown in Figure 5, the normalized phasor vectors can be used to determine the impulse time locations of the ${ZVD}_{F}$ shaper. The angle between ${\bar{v}}_{1}$ and ${\bar{v}}_{2}$ vectors is denoted α. The angle between ${\bar{v}}_{1}$ and ${\bar{v}}_{3}$ vectors is denoted as β.

The α and β using the law of cosines are expressed as

(16) $$\alpha ={cos}^{-1}(\frac{1}{4}\frac{{\omega_{n}}^{4}(4{\tau_{u1}}^{2}{\tau_{u3}}^{2}+{\tau_{u2}}^{2}{\tau_{u3}}^{2}-{\tau_{u1}}^{2}{\tau_{u2}}^{2})+3{\omega_{n}}^{2}{\tau_{u1}}^{2}+5{\omega_{n}}^{2}{\tau_{u3}}^{2}+4}{({\omega_{n}}^{2}{\tau_{u3}}^{2}+1)\sqrt{\left({\omega_{n}}^{2}{\tau_{u1}}^{2}+1)({\omega_{n}}^{2}{\tau_{u2}}^{2}+1\right)}}),$$

(17) $$\beta ={cos}^{-1}(\frac{1}{2}\frac{{\omega_{n}}^{4}({\tau_{u1}}^{2}{\tau_{u2}}^{2}+{\tau_{u2}}^{2}{\tau_{u3}}^{2}-4{\tau_{u1}}^{2}{\tau_{u3}}^{2})-{\omega_{n}}^{2}(3{\tau_{u1}}^{2}+3{\tau_{u3}}^{2}-2{\tau_{u2}}^{2})-2}{({\omega_{n}}^{2}{\tau_{u2}}^{2}+1)\sqrt{\left({\omega_{n}}^{2}{\tau_{u1}}^{2}+1)({\omega_{n}}^{2}{\tau_{u3}}^{2}+1\right)}}).$$

**Figure 6:**
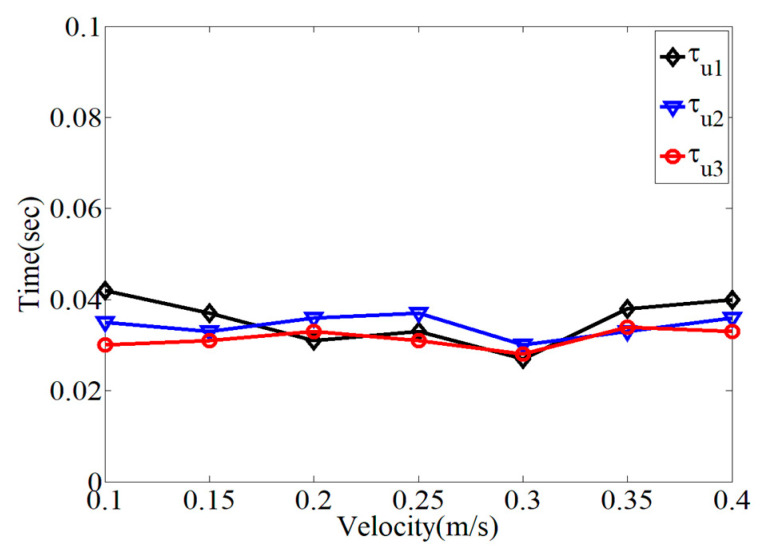
Variation in time constant according to command velocity.

The phasor vectors in Figure 6 and Equations (14) and (15) can be expressed as follows:

(18) $$\theta_{2}=\omega_{n}t_{2}-{tan}^{-1}(\frac{1}{\tau_{u2}\omega_{n}})+{tan}^{-1}(\frac{1}{\tau_{u1}\omega_{n}})=\pi -\alpha ,$$

(19) $$\theta_{3}=\omega_{n}t_{3}-{tan}^{-1}(\frac{1}{\tau_{u3}\omega_{n}})+{tan}^{-1}(\frac{1}{\tau_{u1}\omega_{n}})\;=\pi +\beta ,$$

and impulse times $t_{2}$ and $t_{3}$ can be found using $\theta_{2}$ and $\theta_{3}$, expressed as

(20) $$t_{2}=\frac{1}{\omega_{n}}[\pi -\alpha +{tan}^{-1}(\frac{1}{\tau_{u2}\omega_{n}})-{tan}^{-1}(\frac{1}{\tau_{u1}\omega_{n}})],$$

(21) $$t_{3}=\frac{1}{\omega_{n}}[\pi +\beta +{tan}^{-1}(\frac{1}{\tau_{u3}\omega_{n}})-{tan}^{-1}(\frac{1}{\tau_{u1}\omega_{n}})],$$

The switching times for the stop motion were determined using a formulation with a procedure similar to that of the start motion. $t_{4}$ is denoted as $t_{p}$ and the impulse times $t_{5}$ and $t_{6}$ are expressed as

(22) $$t_{5}=t_{p}+\frac{1}{\omega_{n}}[\pi -\alpha^{\prime }+{tan}^{-1}(\frac{1}{\tau_{d2}\omega_{n}})-{tan}^{-1}(\frac{1}{\tau_{d1}\omega_{n}})],$$

(23) $$t_{6}\;=t_{p}+\frac{1}{\omega_{n}}[\pi +\beta^{\prime }+{tan}^{-1}(\frac{1}{\tau_{d3}\omega_{n}})-{tan}^{-1}(\frac{1}{\tau_{d1}\omega_{n}})],$$

where $\tau_{d1},\;\tau_{d2}$, and $\tau_{d3}$ are selected after $t_{4}$, $t_{5},$ and $t_{6}$, respectively. The angles related to the determination of the impulse time locations are expressed as

$$\alpha^{\prime }={cos}^{-1}(\frac{1}{4}\frac{{\omega_{n}}^{4}(4{\tau_{d1}}^{2}{\tau_{d3}}^{2}+{\tau_{d1}}^{2}{\tau_{d3}}^{2}-{\tau_{d1}}^{2}{\tau_{d2}}^{2})+3{\omega_{n}}^{2}{\tau_{d1}}^{2}+5{\omega_{n}}^{2}{\tau_{d3}}^{2}+4}{({\omega_{n}}^{2}{\tau_{d3}}^{2}+1)\sqrt{\left({\omega_{n}}^{2}{\tau_{d1}}^{2}+1)({\omega_{n}}^{2}{\tau_{d2}}^{2}+1\right)}})$$

$$\beta^{\prime }={cos}^{-1}(\frac{1}{2}\frac{{\omega_{n}}^{4}({\tau_{d1}}^{2}{\tau_{d2}}^{2}+{\tau_{d2}}^{2}{\tau_{d3}}^{2}-4{\tau_{d1}}^{2}{\tau_{d3}}^{2})-{\omega_{n}}^{2}(3{\tau_{d1}}^{2}+3{\tau_{d3}}^{2}-2{\tau_{d2}}^{2})-2}{({\omega_{n}}^{2}{\tau_{d2}}^{2}+1)\sqrt{\left({\omega_{n}}^{2}{\tau_{d1}}^{2}+1)({\omega_{n}}^{2}{\tau_{d3}}^{2}+1\right)}})$$

In (20)–(23), the ${ZVD}_{F}$ shaper for the start and stop commands during the operation of flexible systems with first-order actuators can be expressed as

(24) $$[\begin{array}{c} A_{i}\\ t_{i} \end{array}]=\left[\begin{array}{cccccc} 0.25 & \;\;0.5 & \;\;0.25 & -0.25 & -0.5 & -0.25\\ 0 & \;\;t_{2} & \;\;t_{3} & \;\;t_{p} & \;\;t_{5} & \;\;t_{6} \end{array}\right]$$

Here, (24) can be utilized to produce robust input shaped commands via convolution with arbitrary input commands. From Equations (20) and (21), if $\tau_{u1}$ = $\tau_{u2}$ or $\tau_{u1}$ = $\tau_{u3}$, then the $t_{2}$ or $t_{3}$ of the ${ZVD}_{F}$ shaper become those of the ZVD shaper. Practically, neither case could occur simultaneously because the shaper magnitudes were chosen in a different stepwise fashion. Therefore, the ${ZVD}_{F}$ shaper can be applied in a more general condition, as

(25) $$\tau_{u1}\;\neq \tau_{u2}\;\neq \tau_{u3}$$

In addition, by considering a steady-state error of 2% in (7), for the best deflection reduction performance, the complete command condition of the shaper can be expressed as:

(26) $$t_{i+1}\;\;≻\;t_{i}+\;3\;\tau_{i},$$

where $t_{i}$ is the i-th impulse time and $\tau_{i}$ is the time constant of the i-th impulse. Note that $3\tau_{i}$ is used as the approximate time for the exponential rise or decay to the desired speed at each ramp-up segment or each ramp-down segment.

## 3. Performance Evaluations

In this section, the ${ZVD}_{F}$ shaper is evaluated from the perspective of deflection reduction performance and robustness with respect to time duration ($t_{p}$), time constant ($\tau_{i}$), and system modeling errors. The control performance of the ${ZVD}_{F}$ shaper was compared with that of a ZVD shaper with a pendulum system, as shown in Figure 3.

In Figure 6, the variation in the time constants within each range division was evaluated with respect to the maximum operational velocity. As expected, the time constants in each range division with the ZVD shaper had different values, which were utilized as design references. The time constants have different values depending on the magnitude of the velocity command. For the numerical evaluation of the ${ZVD}_{F}$ shaper, the time constants were selected according to the electrical and physical characteristics of the first-order actuators used in the experimental evaluation, as listed in Table 1. The deceleration time constants ($\tau_{d}$) were set to be the same as the acceleration time constants ($\tau_{u}$).

**Figure 7:**
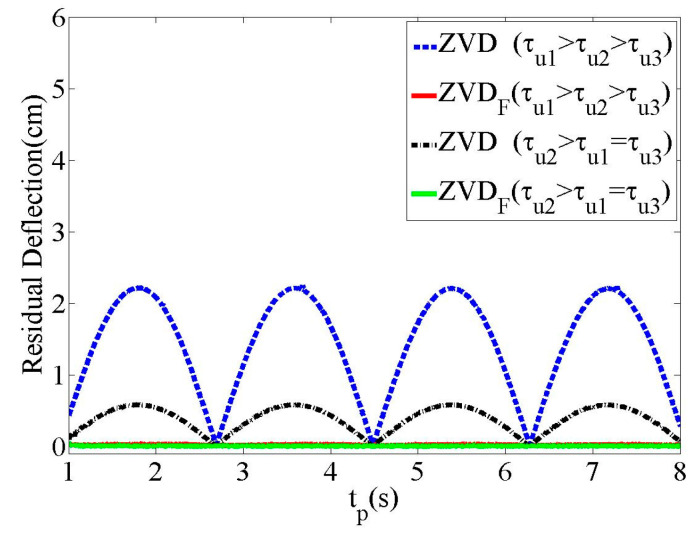
Residual deflection to $t_{p}$.

Figure 7 shows the effectiveness of the residual deflection reduction on the ZVD and ${ZVD}_{F}$ shapers with respect to the duration time ($t_{p}$) with two different time constants, as shown in Table 1. The ZVD shaper produces a large residual deflection for start–stop commands with periodic zero deflection depending on $t_{p}$. Hence, the deflection reduction performance of the ZVD shaper was heavily affected by the variation in the time constants, as expected. However, the ${ZVD}_{F}$ shaper exhibited zero residual deflections regardless of the variations in the time constants.

**Figure 8:**
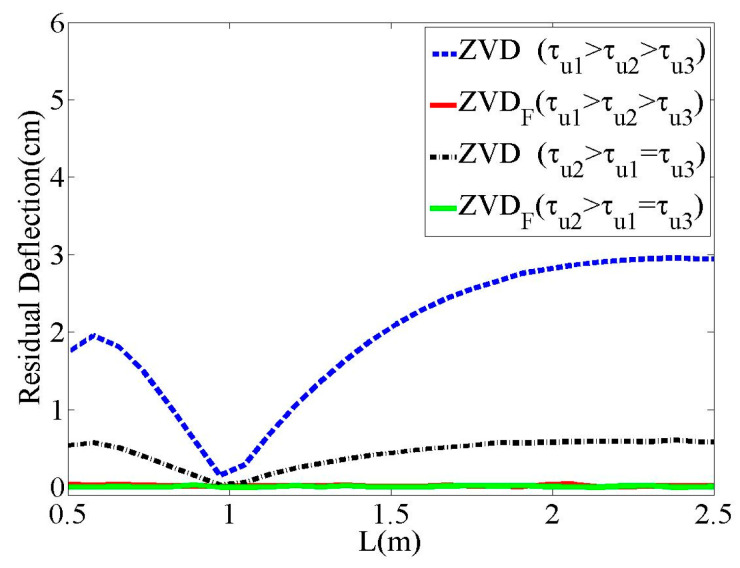
Residual deflection to L.

Figure 8 shows a comparison of the deflection reduction performances of the ZVD and ${ZVD}_{F}$ shapers with respect to the parameter selection, as listed in Table 1. The ZVD shaper produced a large residual deflection with respect to the cable length variation, which was associated with the oscillation frequency. Therefore, the ZVD shaper could not cope with first-order actuators. Therefore, it is necessary for the input shaper design to consider the actuator dynamics. However, the ${ZVD}_{F}$ shaper exhibited almost zero residual deflection regardless of the cable length and time constant throughout the evaluation range.

**Figure 9:**
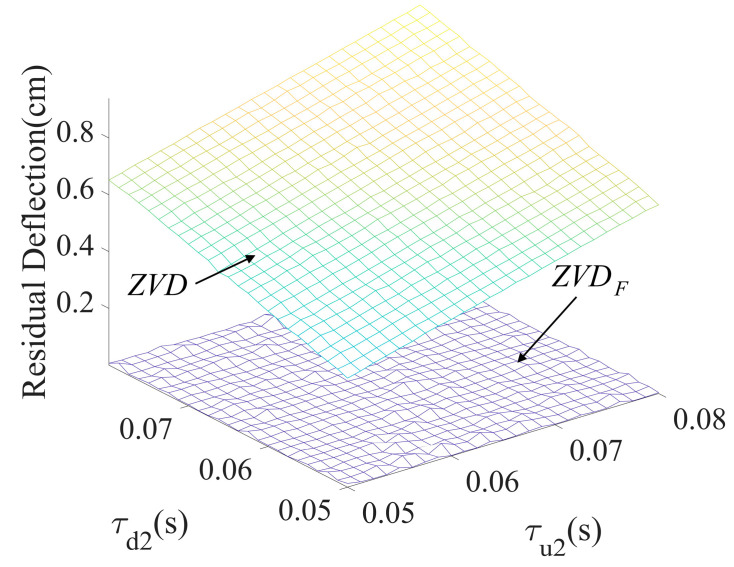
Residual deflection to $\tau_{u2}$ and $\tau_{d2}$.

Figure 9 shows the variational effect of residual deflection as a function of the second acceleration time constant ($\tau_{u2}$) and the second deceleration time constant ($\tau_{d2}$), which produce large amounts of deflection which influences the residual oscillation. The residual reduction performance was evaluated using the parameters listed in Table 1. The ${ZVD}_{F}$ shaper showed a much better control performance than the ZVD shaper throughout the evaluation range of the time constants.

**Figure 10:**
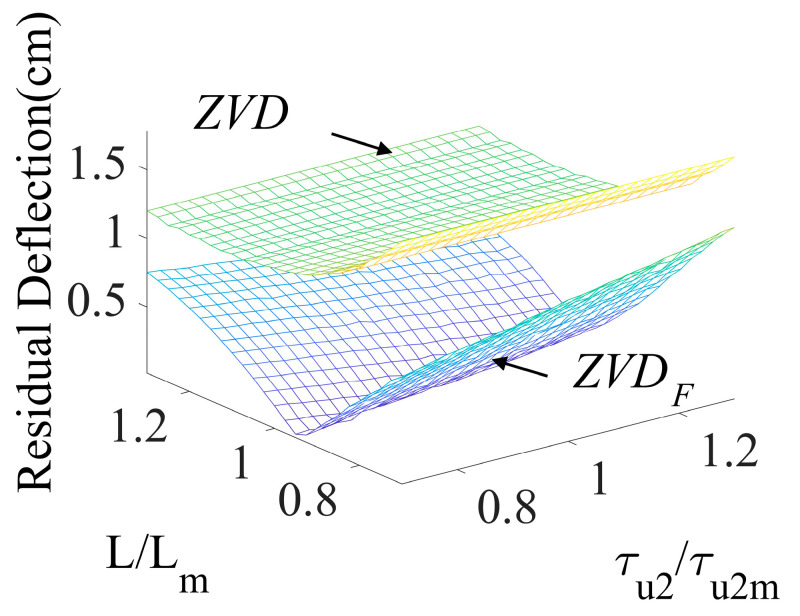
Sensitivity to system and actuator modeling errors.

Figure 10 shows the robustness of the ${ZVD}_{F}$ and ZVD shapers with respect to the cable length and second acceleration time constant. Neither shaper affected the variation in the time constant. But the ${ZVD}_{F}$ shaper presented more robustness than the ZVD shaper, which had large residual deflection in the performance comparison of both shapers. The ${ZVD}_{F}$ shaper showed a better residual deflection reduction performance than the ZVD shaper throughout the variation range of ±20% second time constant ($\tau_{u2}$).

**Figure 11:**
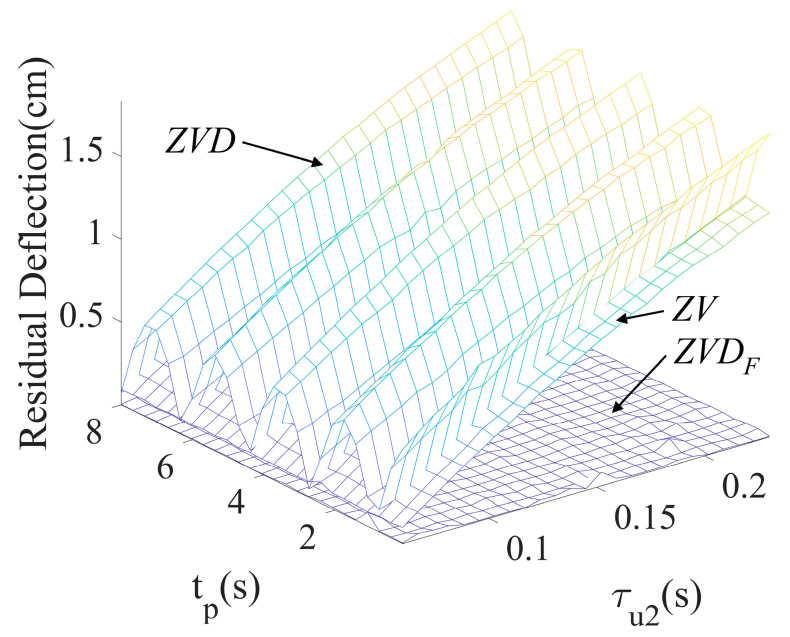
Residual deflection ${ZVD}_{F}$, ZVD, and ZV shapers to $\tau_{u2}$ and $t_{p}$.

Figure 11 shows the comparison of residual deflection reduction performance as a function of the first acceleration time constant ($\tau_{u2}$) and duration time ($t_{p}$) with resepct to the ${ZVD}_{F}$, ZVD, and ZV shapers. The ZV shaper is briefly summarized as follows [5]:

(27) $$[\begin{array}{c} A_{i}\\ t_{i} \end{array}]=\left[\begin{array}{cc} \frac{1}{K+1} & \frac{K}{K+1}\\ 0 & ∆T \end{array}\right],\;i=1,2$$

where ∆T and K are the same symbols as in (1). The ZVD shaper showed periodical residual deflection according to duration time, as shown in Figure 7, and produced a large residual deflection as $\tau_{u2}$ increased. The ZV shaper could not cope with large time constants even if it is acceptable with small time constants. On the other hand, the ${ZVD}_{F}$ shaper presented no residual deflection regardless of variations in $\tau_{u2}$ and $t_{p}$.

**Figure 12:**
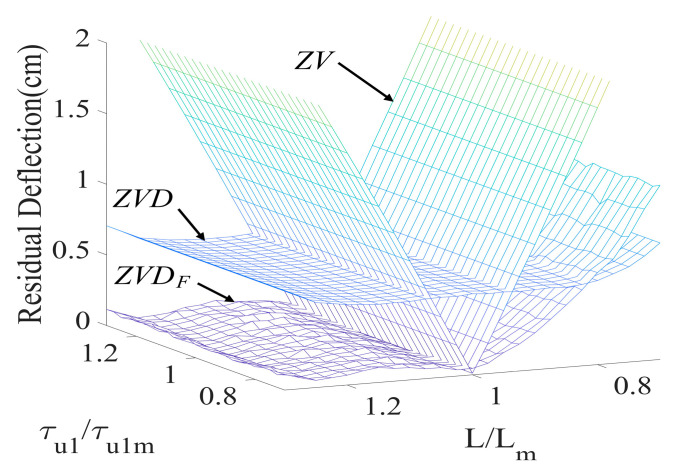
Sensitivity of ${ZVD}_{F}$, ZVD, and ZV shapers to $\tau_{u1}$ and L.

Figure 12 presents the sensitivity of the ${ZVD}_{F}$, ZVD, and ZV shapers as a function of cable length ($L_{m}$) and the first acceleration time constant ($\tau_{u1}$) to evaluate the effect of modeling error and initial command modification. The ZVD shaper exhibited a large residual deflection, owing to the collapse of the input command by the first-order actuators. However, the ${ZVD}_{F}$ shaper appropriately coped with the modeling error and produced a small residual deflection in the evaluation range.

According to the numerical evaluations described in Section. 3, the ${ZVD}_{F}$ shaper had a better robustness and residual deflection reduction performance than the conventional ZVD shaper under input command distortion, owing to the first-order actuators. The ZV shapers do not have any effect on the variation in the time constant of first-step commands and produces a residual deflection with respect to modeling errors. As a result, to control the performance under first-order actuators, nonlinear dynamic effects needed to be accommodated when designing an input shaping technique. In the next section, the ${ZVD}_{F}$ shapers are experimentally tested with a mini bridge crane with respect to modeling errors.

## 4. Experimental Verification

**Figure 13:**
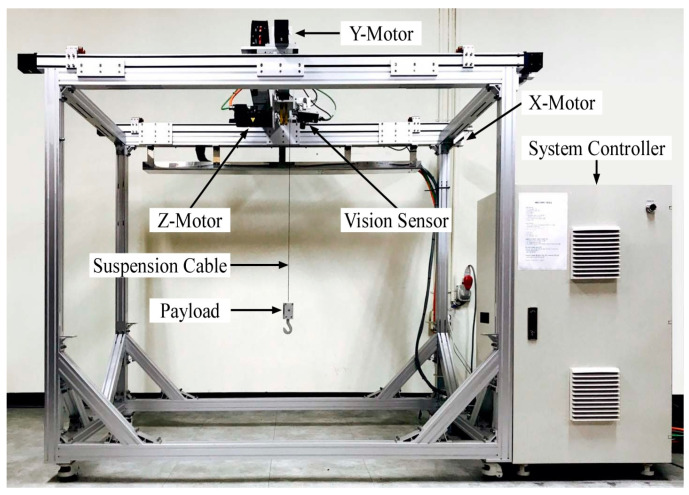
Mini bridge crane.

**Figure 14:**
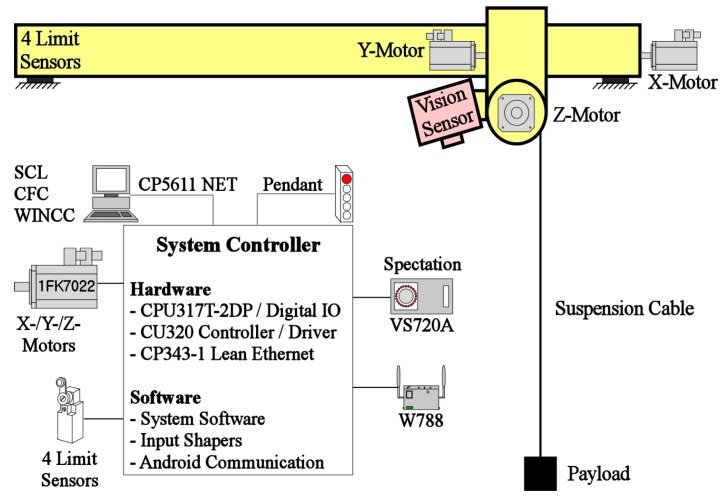
Hardware configuration.

To experimentally validate the performance of the proposed input shaper shown in the previous section, a mini bridge crane, which has the dimensions of 1.3 m (length) × 0.75 m (width) × 1.5 m (height), is utilized, as shown in Figure 13. The hardware and software components are illustrated in Figure 14. In the hardware component, a Siemens programmable logic controller (PLC) was installed and programmed using a personal computer to verify the proposed input shaper. The PLC processor was connected to a Senamics motor (Siemens, Seoul, Republic of Korea) driven by an Ethernet module. The mini bridge crane was operated using three synchronous AC motors and four proximity sensors. The software components were configured using CFC, SCL, and WinCC for system operation and experimental data processing. A Spectation^®^ program for VS720 (Siemens, Seoul, Republic of Korea) series vision sensor is utilized for the deflection measurement of a payload.

**Figure 15:**
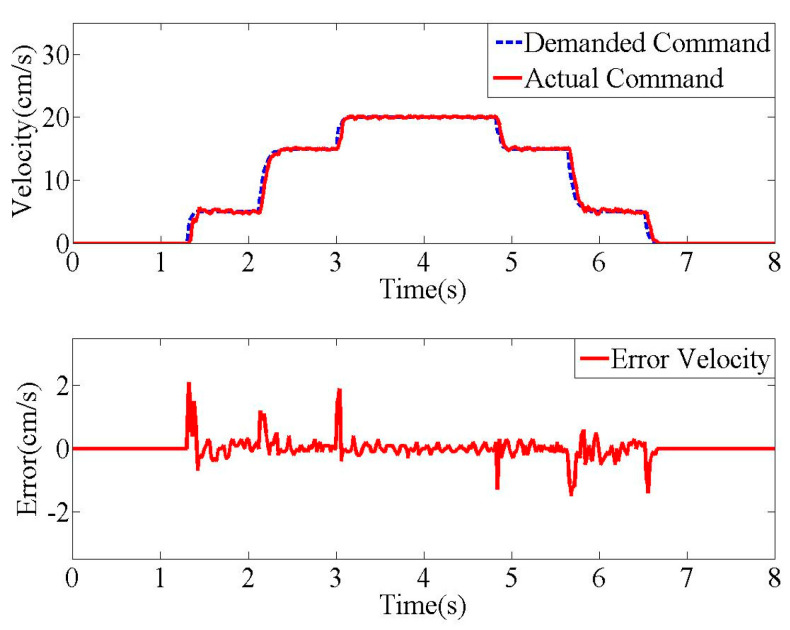
Experimental command accuracy.

For the experimental evaluation of input shaped commands, the operational system was set to track the desired commands distorted by first-order actuators as accurately as possible. The proportional gain and integral gain on the Sinamics driver were 0.25 and 10 ms, respectively. Figure 15 shows that the desired and actual velocity commands are closely executed within a ±2 cm/s error range. In this experiment, the input shaper parameters were selected, as listed in Table 1. Hence, the system settings could be used to validate various input shapers with first-order actuators.

**Figure 16:**
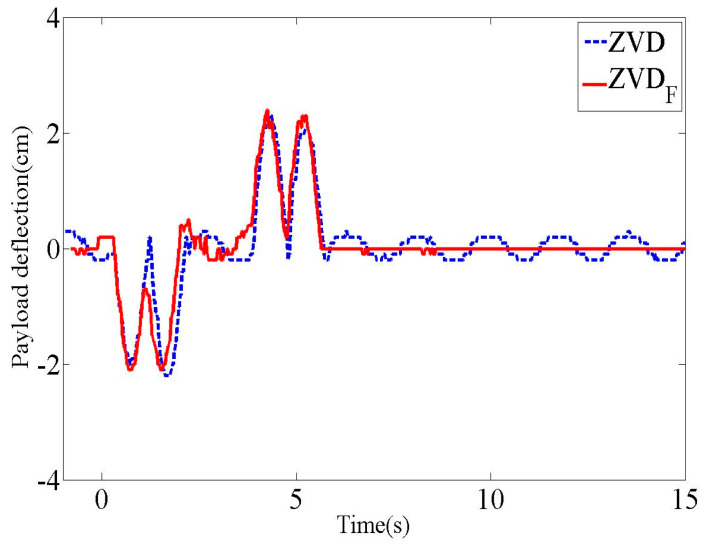
Payload deflection responses of ZVD and ${ZVD}_{F}$ shaper.

As a fundamental experimental evaluation of the control performance of the input shapers listed in Table 1 and the above system settings, the residual deflection reduction performances are presented in Figure 16. In the transient period, there were small deflection magnitudes for both input shapers. However, the ZVD shaper exhibited an oscillatory deflection, unlike the ${ZVD}_{F}$ shaper, which had zero residual deflection after the end of the input commands. It is clear that the ${ZVD}_{F}$ shaper has a better residual deflection reduction performance than the ZVD shaper for first-order actuators.

**Figure 17:**
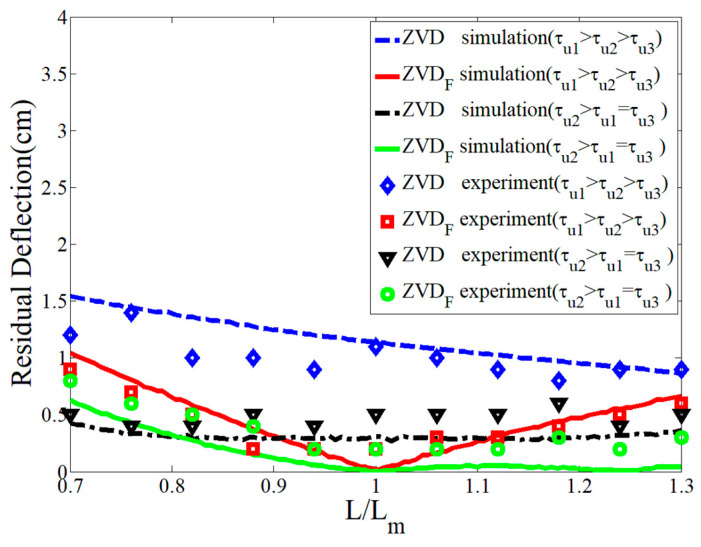
Sensitivity of ${ZVD}_{F}$ and ZVD shapers to $L/L_{m}$.

Figure 17 presents an experimental robustness comparison of the ${ZVD}_{F}$ and ZVD shapers as a function of the modeling errors with different combinations of the time constants of the first-order actuators, as shown in Table 1. As indicated in the previous section, the ZVD shaper shows large residual deflection even if it shows relatively low residual deflection at $\tau_{u2}\;>\tau_{u1}=\tau_{u3}$ regardless of the range of modeling errors. However, the ${ZVD}_{F}$ shaper was more robust than the ZVD shaper throughout the evaluation range of the cable length ratio ($L/L_{m}$) with zero residual deflection at $L/L_{m}=1$, meaning there was no error in the system modeling. As expected, the ${ZVD}_{F}$ shaper could cope with the time constant variation of the first-order actuators better than the ZVD shaper.

**Figure 18:**
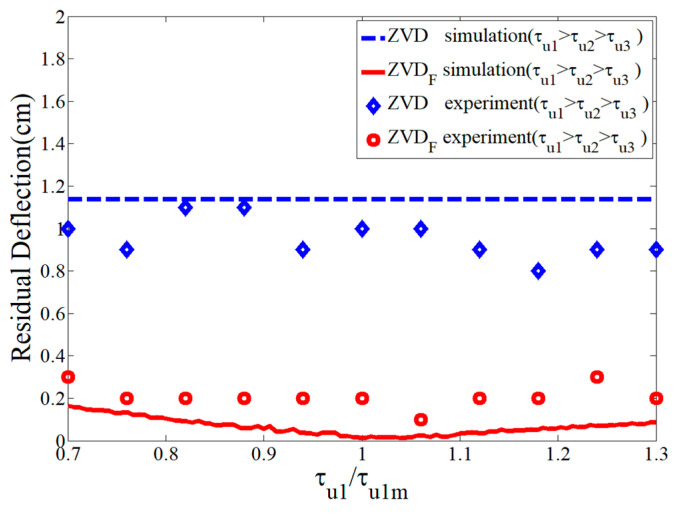
Sensitivity of ${ZVD}_{F}$ and ZVD shapers to $\tau_{u1}/\tau_{u1m}$.

Figure 18 compares the robustness of the ${ZVD}_{F}$ and ZVD shapers as a function of the first time constant. The ZVD shaper produced a large residual deflection; therefore, it had low robustness, which is largely affected by the time constant. However, the ${ZVD}_{F}$ shaper showed a better robustness with respect to the system modeling error than the ZVD shaper. From the experimental validation, the design process for the input shaping technique should reveal the inclusion of first-order actuator dynamics. In the experimental data presentation, it was difficult to execute and obtain precise data points in small quantities as they were easily affected by structural rigidity and unknown non-ideal effects.

**Figure 19:**
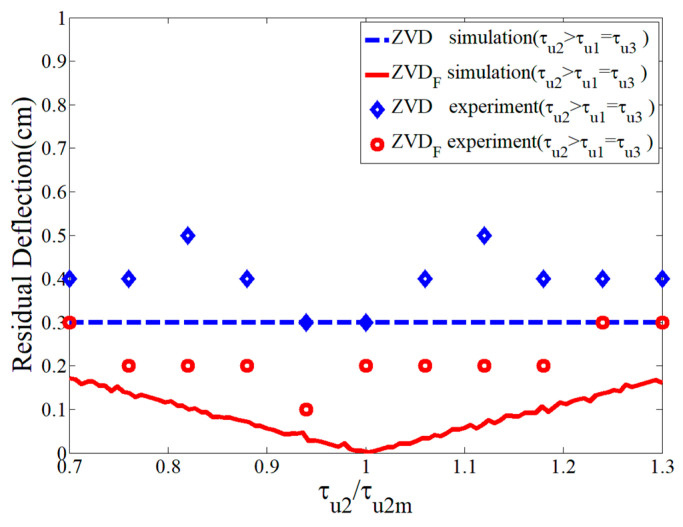
Sensitivity of ${ZVD}_{F}$ and ZVD shapers to $\tau_{u2}/\tau_{u2m}$.

Figure 19 shows a robustness comparison of the ${ZVD}_{F}$ and ZVD shapers as a function of the second acceleration time constant. The ZVD shaper had a constant residual deflection regardless of the modeling error of the time constant. Even if there were some measurement errors because of small deflection quantities, the ${ZVD}_{F}$ shaper showed a better residual deflection reduction performance than the ZVD shaper.

## 5. Conclusions

A robust input shaper with first-order actuators was proposed using the phase vector approach. An analytical solution was developed by employing an exponential approximation function and the conventional shaper magnitude for a simplified derivation. The robust input shaper was numerically and experimentally validated by evaluating the residual deflection reduction performance and parameter sensitivity of the model system as a counterpart to a conventional robust input shaper. Throughout the theoretical and experimental verifications, the proposed robust input shaper clearly presented a much better control performance with a first-order actuator under parameter uncertainties than the conventional robust input shaper did.

## Figures and Tables

**Table 1:** System parameters for ${ZVD}_{F}$ shaper evaluation.

L	$\tau_{u1}$	$\tau_{u2}$	$\tau_{u3}$	$V_{d}$	$t_{p}$
0.8 m	0.03 s	0.06 s	0.03 s	0.2 m/s	3.5 s

## Data Availability

The original contributions presented in the study are included in the article, further inquiries can be directed to the corresponding author.
